# Multimodal Imaging Technology Effectively Monitors HER2 Expression in Tumors Using Trastuzumab-Coupled Organic Nanoparticles in Patient-Derived Xenograft Mice Models

**DOI:** 10.3389/fonc.2021.778728

**Published:** 2021-11-17

**Authors:** Li Wen, Lei Xia, Xiaoyi Guo, Hai-Feng Huang, Feng Wang, Xian-teng Yang, Zhi Yang, Hua Zhu

**Affiliations:** ^1^ Medical College, Guizhou University, Guiyang, China; ^2^ Key Laboratory of Carcinogenesis and Translational Research (Ministry of Education/Beijing), NMPA Key Laboratory for Research and Evaluation of Radiopharmaceuticals (National Medical Products Administration), Department of Nuclear Medicine, Peking University Cancer Hospital & Institute, Beijing, China; ^3^ Department of Orthopaedics, Guizhou Provincial People’s Hospital, Guiyang, China

**Keywords:** trastuzumab, dMNPs, PET/MRI imaging, HER2, micro-PET/CT imaging

## Abstract

Trastuzumab is a monoclonal antibody targeting human epidermal growth factor 2 (HER2), which has been successfully used in the treatment of patients with breast cancer and gastric cancer; however, problems concerning its cardiotoxicity, drug resistance, and unpredictable efficacy still remain. Herein, we constructed novel organic dopamine–melanin nanoparticles (dMNs) as a carrier and then surface-loaded them with trastuzumab to construct a multifunctional nanoprobe named Her-PEG-dMNPs. We used micro-PET/CT and PET/MRI multimodality imaging to evaluate the retention effect of the nanoprobe in HER2 expression in gastric cancer patient-derived xenograft (PDX) mice models after labeling of the radionuclides ^64^Cu or ^124^I and MRI contrast agent Mn^2+^. The nanoprobes can specifically target the HER2-expressing SKOV-3 cells *in vitro* (3.61 ± 0.74 vs. 1.24 ± 0.43 for 2 h, *P *= 0.002). *In vivo*, micro-PET/CT and PET/MRI showed that the ^124^I-labeled nanoprobe had greater contrast and retention effect in PDX models than unloaded dMNPs as carrier (1.63 ± 0.07 vs. 0.90 ± 0.04 at 24 h, *P *= 0.002), a similarity found in ^64^Cu-labeled Her-PEG-dMNPs. Because ^124^I has a longer half-life and matches the pharmacokinetics of the nanoparticles, we focused on the further evaluation of ^124^I-Her-PEG-dMNPs. Furthermore, immunohistochemistry staining confirmed the overexpression of HER2 in the animal model. This study developed and validated novel HER2-specific multimodality imaging nanoprobes for quantifying HER2 expression in mice. Through the strong retention effect of the tumor site, it can be used for the promotion of monoclonal antibody treatment effect and process monitoring.

## Introduction

Human epidermal growth factor was discovered in 1962, while structurally related receptors were discovered in 1978 (1). As for the HER family, it is mainly composed of four receptors, HER1, HER2, HER3, and HER4, which can interact with each other in regulating different biological behaviors such as cell proliferation, differentiation, and survival. Human epidermal growth factor 2 (HER2) is a transmembrane glycoprotein, also known as CERBB-2 and ERbb2, which promotes malignant biological behaviors such as cell proliferation and migration through the formation of homologous or heterologous dimers with family members (2). Amplification of the HER gene and overexpression of its products were found for the first time in breast cancer (3, 4). Positive HER2 expression was found in 15%–20% of breast cancers, which had a high degree of malignancy and poor prognosis (4). HER2 is an ideal molecular target for cancer, highly expressed in a variety of cancer cells, such as colorectal cancer, ovarian cancer, prostate cancer, bladder cancer, and lung cancer, especially gastric and esophageal cancer (3–6).

According to “Cancer Statistics, 2020”, there were approximately 19.3 million new cancer cases and 10.0 million cancer deaths in the world (7). Gastric cancer has been one of the leading causes of common cancer and cancer-related deaths. Trastuzumab (Herceptin), a recombinant humanized IgG monoclonal antibody, binds to the extracellular domain IV of HER2 for antitumor activity and was approved by the US Food and Drug Administration as first-line treatment of advanced HER2-positive gastric cancer (8). HER2 was overexpressed in 7%–34% of gastric cancer patients and has been demonstrated to increase overall survival in locally advanced or metastatic gastric cancer (9, 10). At present, many researchers have reported ^68^Ga, ^18^F, ^89^Zr, ^64^Cu, and other positron nuclide-labeled PET molecular probes targeting HER2, for example, monoclonal antibody fragments, affibody, and nanobody (11–16). Preliminary clinical trials have been conducted studying ^64^Cu/^89^Zr-trastuzumab, ^89^Zr-pertuzumab, ^68^Ga-nanobody, and so on (14, 17–19). However, some probes still have problems such as drug resistance and toxic side effects (17, 20). In addition, intratumoral heterogeneity and small biopsy tumor samples may not represent HER2 expression across the tumor or metastatic tumor lesions (6, 21). Therefore, a more accurate, non-invasive HER2 status assessment is needed to help guide physicians to tailor treatment for each patient, monitor HER2-targeted therapy responses, and identify those patients who develop resistance.

Multimodal tumor molecular imaging is a hot spot in medical imaging research. By constructing specific molecular probes for tumor cell surface receptors and combining the functions of different imaging contrast agents to provide multiple imaging effects, collaborative imaging that cannot be matched by single imaging can be obtained, which has a good clinical application prospect in the early diagnosis of diseases. In view of the problems existing in the application of trastuzumab, nanomedicine based on molecular imaging can provide a direction for solving such concerns. The hydrodynamic properties of antibodies will be changed by combining monoclonal antibodies with high-quality nanoparticles, which can improve the tumor uptake and retention rate and reduce heart toxicity (22, 23). On the other hand, the nanoprobe can load highly sensitive imaging molecules to achieve multimodality imaging function, which can be used to monitor the treatment process in real time and adjust the individualized treatment plan. This is also a major trend of precision medicine in the future (23). Nanoparticle-based probes enable non-invasive imaging of proteolytic activity for cancer diagnosis. However, there are also some toxic problems in the application of nanoparticles. The exogenous inorganic nanoparticles not only provide a strong contrast, but also have high sensitization and high toxicity and are difficult to remove *in vivo*, which cannot be ignored (24–26). The emergence of organic nanoparticles based on endogenous biological materials increases the application scope and safety of nanotechnology and has been widely studied in the field of molecular imaging (26, 27).

Melanin is a ubiquitous, amorphous, and irregular biopolymer, which is widely distributed in almost all living organisms and has many unique functions (28, 29). Dopamine–melanin nanoparticles (dMNPs) consist of naturally producing dopamine with good biocompatibility and biodegradability (30). It retains many characteristics of melanin and can directly complex with various metal ions without chelating agents, which significantly simplifies the preparation process and reduces the heterogeneity of the nanoparticles. dMNPs have been demonstrated to be used for tumor multimodality imaging, such as photoacoustic imaging (PAI), positron emission tomography (PET), and magnetic resonance imaging (MRI) (27, 31). This study reports the characterization, quality control, and micro-PET/CT and PET/MRI multimodality imaging evaluation of a novel nanoprobe—(^124^I/^64^Cu, Mn)-Her-PEG-dMNPs—and applied this probe in gastric cancer patient-derived xenograft (PDX) models. The results indicate that the novel nanoprobe Mn-Her-PEG-dMNP has a good retention effect on tumors and has great potential in the treatment of gastric cancer.

## Materials and Methods

### Materials

Dopamine hydrochloride, MnCl_2_, and ammonia aqueous solution were obtained from Sigma-Aldrich (St. Louis, MO, USA). Amine-mPEG5000 (mPEG5000–NH_2_, 5,000 Da) was acquired from Aladdin (Shanghai, China). Herceptin was obtained from Roche Pharmaceuticals (Shanghai, China) Ltd. 2-Mercaptoethanol was purchased from Sinopharm Chemical Reagent Co. Ltd. For the cell experiments, Dulbecco’s modified Eagle’s medium (DMEM) and fetal bovine serum (FBS) were purchased from Gibco (USA); penicillin–streptomycin mixture double and anti-0.25% trypsin (including EDTA) were purchased from Invitrogen (USA). In the experiment, high nuclear pure simple ^124^I was prepared by ^124^Te solid target of HM-20 cyclotron at the Nuclear Medicine Department of Peking University Cancer Hospital. The nuclide was purified by ^124^I special purification device and collected in 0.02 M NaOH solution (radioactive concentration 148 MBq/ml). The positron nuclide ^64^Cu was prepared by bombarding a ^64^Ni solid target with a medical cyclotron at the Nuclear Medicine Department of Peking University Cancer Hospital with 3.7 MBq/μl in 0.01 M HCl solution (32).

### Synthesis and Modification of dMNPs

Dopamine–melanin nanoparticles were prepared according to the methods reported in previous literature (30). Briefly, ammonia aqueous solution (0.3 ml, NH_4_OH, 28%–30%) was mixed with ethanol (4 ml) and deionized water (9 ml) under mild stirring at 40°C for 30 min. Fifty milligrams of dopamine hydrochloride was dissolved in water (1 ml), then injected into the above mixture solution, and stirred for 48 h. The color of this solution changes from light yellow to dark brown. The bright black solution was then centrifuged with a centrifugal filter (Amicon centrifugal filter device, MWCO = 50 kDa) and washed several times to remove the unpolymerized dopamine–melanin molecules. The reaction solution was taken and diluted to 5 ml ultrapure water by ultrasonication for 5 min, and the reaction system pH = 9 was adjusted by NH_4_OH (25%–28%). Then, the solution was slowly dripped into the reaction bottle containing 25 mg diamino polyethylene glycol (NH_2_–PEG_5000_–NH_2_) and stirred at room temperature for 12 h. The unreacted NH_2_–PEG_5000_–NH_2_ was removed by a 50-kDa ultrafiltration centrifuge to obtain NH_2_–PEG-dMNPs.

### Preparation of the Her-PEG-dMNPs and Mn-Her-PEG-dMNPs

The amino-sulfhydryl cross-linking agent Sulfo-SMCC was used as a linker in the combination of Herceptin-SH and NH_2_–PEG-dMNPs. Five milligrams of NH_2_–PEG-dMNPs (0.5 × 10^−4^ mM) was fully dispersed in 1 ml phosphate buffered solution (PBS), and excess amino-sulfhydryl cross-linking agent Sulfo-SMCC (5 µM) was stirred at room temperature for 2 h. Then, the unreacted Sulfo-SMCC was separated by the PD-10 column. The reaction solution was concentrated to 0.6 ml by ultrafiltration and slowly added to Herceptin-SH (0.33 × 10^−4^ mM). After reacting for 2 h at room temperature with stirring, the unreacted sulfhydryl–Herceptin was removed using a PD-10 column (GE Healthcare, USA) with an eluent of PBS (pH = 7.4) and lyophilized to obtain a purified Her-PEG-dMNP. Two milligrams of Her-PEG-dMNPs and MnCl_2_ (10 × 10^−3^ mM) were fully dispersed in 1 ml deionized water and stirred at room temperature for 2 h. The Mn-Her-PEG-dMNPs were obtained by centrifugation with an ultrafiltration centrifuge tube (MWCO = 10 kDa).

### Characterization

The morphology of Her-PEG-dMNPs was characterized by using a JEM-2100F transmission electron microscope with an accelerating voltage of 200 kV, and the sample was made by using a film of copper mesh. Dynamic light scattering (DLS) was used to detect the hydrodynamic diameter of the dMNPs, PEG-dMNPs, and Her-PEG-dMNPs with a laser light-scattering spectrometer (SLS-5022F, Germany) ^1^H-NMR spectra of dMNPs and PEG-dMNPs were detected by a 600-MHz NMR (Bruker) spectrometer with D_2_O as the solvent.

### Cytotoxicity Assay

The viability and proliferation of HeLa and HepG2 cells were evaluated by a standard 3-(4,5-dimethylthiazol2-yl)-2,5-diphenyltetrazolium bromide (MTT) assay. Typically, HeLa and HepG2 cells were incubated in the culture medium at 37°C in an atmosphere of 5% CO_2_ and 95% air for 24 h. Then, the medium was removed. The cells were incubated in cultured medium containing Her-PEG-dMNPs and Mn-Her-PEG-dMNPs with different concentrations of 0.125, 0.25, 0.5, 1, and 2 mg/ml for 24 h and washed with the medium twice. The MTT solution (20 µl, 0.5 mg/ml) was added to each well, and the cells were incubated for 4 h without light at 37°C. After removal of the supernatant, the residues were lysed with 150 µl of dimethyl sulfoxide (DMSO). Finally, the light absorption (OD) values were measured at 490 nm with a microplate reader. The untreated cells were processed analogously, and their OD values were used as the reference to calculate 100% cellular viability.

### Radiosynthesis and Quality Control of the Radiotracer

^64^Cu was diluted with hydrochloric acid (0.01 M, 300 µl), then a small amount of sodium acetate (0.1 mol/L) was added to adjust the pH to 5.5, and then Her-PEG-dMNP (2 mg/ml) was added and incubated at 40°C for 1 h. When the reaction was over, saturated EDTA solution (0.05 M) was added and mixed with the reaction liquid. ^64^Cu-Her-PEG-dMNP was purified using PD-10 column in PBS (pH = 7.4). ^124^I can label Her-PEG-dMNPs *in situ* using N-bromosuccinimide as the oxidant. The ^124^I buffer was diluted with PB solution (0.1 M, 500 µl) before being added to the Mn-Her-PEG-MNPs. Then, 20 µl N-bromosuccinimide (NBS, 5.6 mM) was added to the above solution for 1 min. The radiolabeled reaction was then ended and the ^124^I-Her-PEG-dMNP was achieved *via* the PD-10 column in PBS buffer, and radio-TLC was used to detect the radiolabeling rate of the ^124^I-Her-PEG-dMNPs with acetone as the developer. The stability of the ^64^Cu-HER-PEG-dMNPs and ^124^I-Her-PEG-dMNPs *in vitro* was measured by incubations in PBS (pH = 7.4) and 5% HSA solution. Radio-TLC was used to analyze the radiochemical purity of the ^124^I/^64^Cu-Her-PEG-dMNPs at different time points.

### *In-Vitro* Cell Uptake

One day before the study, human ovarian carcinoma SKOV-3 cells were seeded in 24-well plates at an average of 1 × 10^5^ cells per well and incubated overnight in 1 ml of DMEM medium. Prior to the study, the cells were washed twice with 1 ml of cold PBS, and after the culture solution was aspirated in each well, the cells were re-incubated with 500 μl of serum-free medium for 2 h at 37°C. Then, four wells in every dish were divided into one group. A total of 37 kBq of ^124^I/^64^Cu-Her-PEG-dMNPs in 500 µl serum-free medium was added to each well, and the cells were cultured at 37°C with 5% CO_2_. To assess the specific targeting ability of ^124^I/^64^Cu-Her-PEG-dMNPs for targeting HER2, three groups of SKOV-3 cells (*n* = 4) were co-incubated with ^124^I/^64^Cu-HER-PEG-dMNPs and 20 µg of Her-PEG-dMNPs. After co-incubation with the radiotracer for 10, 30, 60, and 120 min, the cell culture media of each group was sequentially aspirated, and the cells were rinsed three times with 1 ml of cold PBS and lysed with 500 µl of 0.5 M NaOH. Cell lysates in each well were then collected and counted by a γ-counter.

### Subcutaneous Tumor Models

All animal procedures were performed in accordance with the Guidelines for the Care and Use of Laboratory Animals of Peking University (Beijing, China), and experiments were approved by the Animal Ethics Committee of the National Regulation of China for the Care and Use of Laboratory Animals and in compliance with the guidelines established by Peking University Cancer Hospital Animal Care and Use Committee. The PDX models were constructed as in reference (11). Fresh tumor tissue mass derived from patients was inoculated into the right groin area of NOD-SCID mice, and the tumor tissue was removed when the tumor grew to 1 cm in diameter. The tissue blocks were cut into 0.05 cm * 0.05 cm * 0.05 cm for subculture.

### Pharmacokinetics Studies

Healthy KM mice (18–20 g) were used to conduct the pharmacokinetics study. Mice were sealed off 3 days in advance with 0.5% potassium iodide (KI), and ^124^I-Her-PEG-dMNP (3.7 MBq) was intravenously injected into each mouse (*n* = 5) at set intervals (1, 3, 5, 10, 15, 30, 60, and 120 min and 4, 8, 24, 48, and 72 h). Then, ^64^Cu-Her-PEG-dMNP also was intravenously injected into each mouse (*n* = 5) at set intervals (1, 3, 5, 10, 15, 30, 60, and 120 min and 4, 8, 24, and 48 h) and into the blood through the orbital vein. The blood samples were weighed before counting using a γ-counter. The results were calculated as the percentage of injected dose per gram (%ID/g, mean ± SD), and the parameters of the pharmacokinetics were analyzed using the GraphPad Prism software.

### Micro-PET/CT Imaging Studies in PDX NOD-SCID Mice

Micro-PET/CT imaging studies were conducted by using PDX model NOD-SCID mice with a micro-PET/CT system (Super Nova PET/CT, PINGSENG, Shanghai, China). Tumor-bearing NOD-SCID mice were injected *in situ* with ^124^I-Her-PEG-dMNPs and ^124^I-PEG-dMNPs (1.11 MBq). Anesthesia for the animals was performed using a PINGSENG isoflurane gas anesthesia system. Axial and coronal PET/CT images of the tumor were collected at 2, 24, 48, and 72 h. The mice were anesthetized with isoflurane before imaging, and anesthesia was maintained during imaging (volume coefficient 1%). Micro-PET images were acquired for 15 min and reconstructed with attenuation correction based on the CT data (CT–AC reconstruction). Regions of interest (ROIs) were drawn on the CT images and further mapped on PET. The experiments were repeated three times at each time point.

### PET/MRI of the Mn-Her-PEG-dMNPs

For *in-vivo* MRI, PDX model NOD-SCID mice (*n* = 3) were first anesthetized by intraperitoneal injection of 4% chloral hydrate solution (50 mg/kg), when the tumors reached approximately 0.8 cm, followed by *in-situ* injection of Mn-Her-PEG-dMNPs (0.125 mM, 30 µl/mouse). Axial images of tumor locations were collected at 2, 24, and 72 h, using a clinical 1.5-T magnetic resonance scanner (Siemens) with animal microcoils. The MRI sequence was as follows: TR, 531 ms; TE, 9.1 ms; flip angle, 30°; FOV, 160 × 100 mm^2^; matrix, 256 × 256; and slice thickness, 3 mm. The analysis of the PET/MRI signal in the tumor images was conducted with the 3D Slicer software.

### Immunohistochemistry of PDX Tumor Tissues

Mice were sacrificed after the last imaging point, and then the tumor tissue was immediately removed and fixed in formalin. For immunohistochemical staining, sections of PDX tumor tissue were placed in a beaker containing citrate antigenic repair solution. Intermittent heating was performed three times with microwaves. Then, the tissue was treated with 10% goat serum at 37°C for 30 min. After being rinsed three times with PBS, the samples were incubated overnight with anti-HER2 antibody (1:300) at 4°C, and then sections were incubated with biotinylated goat anti-rabbit (HRP) antibody (1:1,000) at 37°C for 30 min. The sections were then stained with DAB substrate kit and hematoxylin and differentiated with 0.25% hydrochloric alcohol. Sections were dehydrated and sealed with neutral gum and photographed for analysis.

## Results

### Synthesis and Characterization of the HER-PEG-dMNPs

**Scheme 1:**
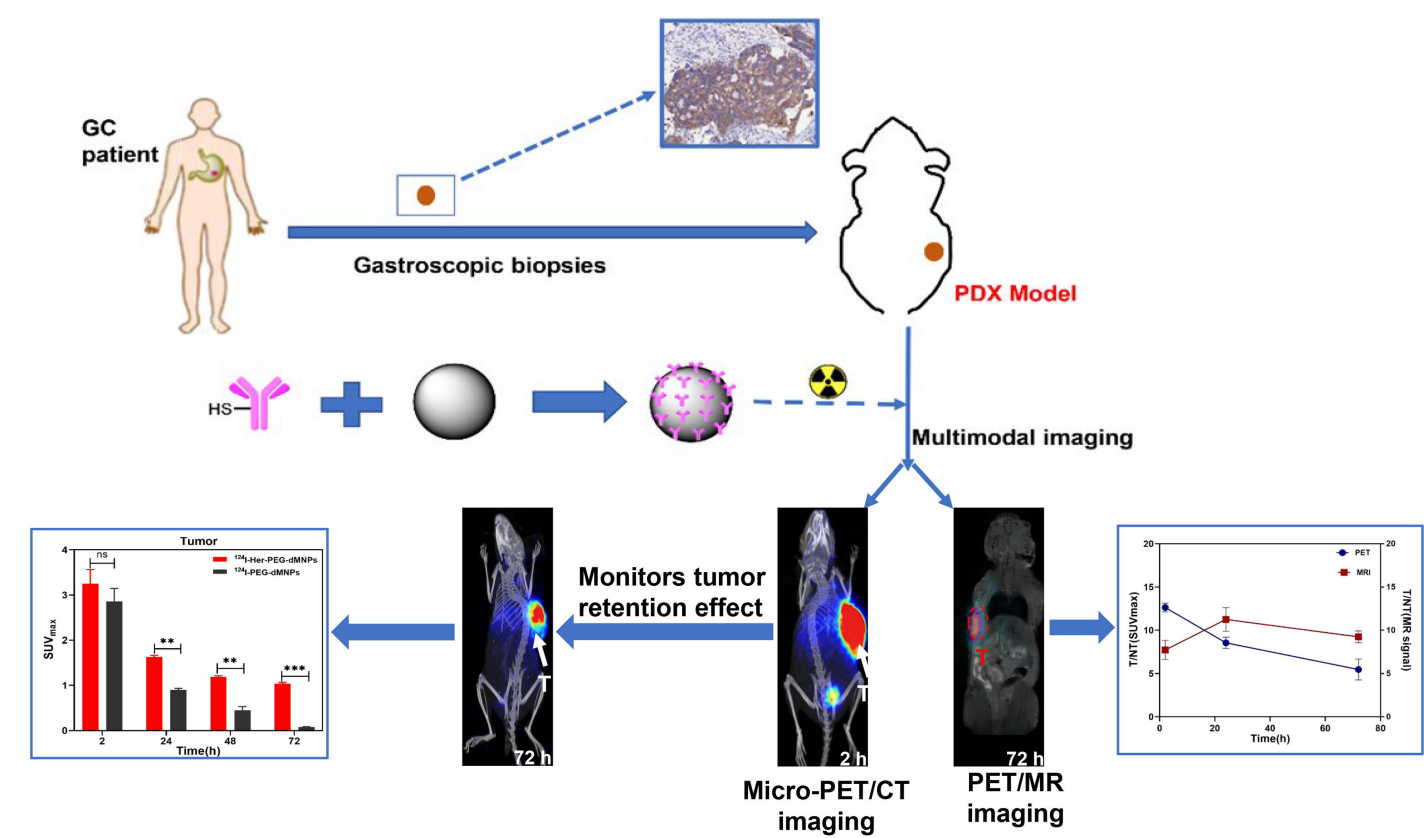
Synthesis and characterization of ^124^I/^64^Cu-Mn-Her-PEG-MNPs. ^124^I, ^64^Cu, and Mn^2+^were used to construct a tumor special nanoprobe for MRI/PET imaging of the gastric cancer PDX model. All the numerical data are presented as mean ± SD. *P < 0.05; **P < 0.01; ***P < 0.001 by two-way ANOVA. The symbols **, *** indicates statistically significant, very statistically significant, and the expanded form of ns indicates no statistically significant.

**Figure 1:**
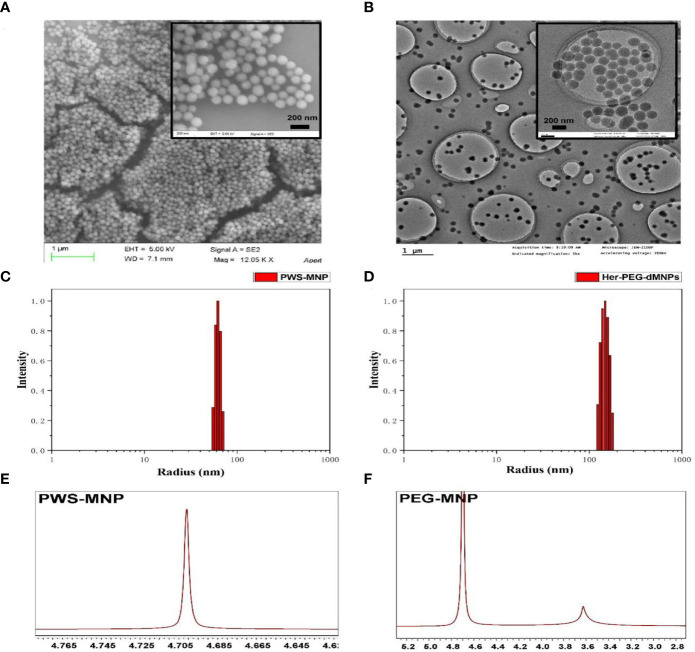
Characterization of the nanoprobe. **(A)** SEM of dMNPs with a scale bar = 1 µm, and Her-PEG-dMNPs (reduction) with a scale bar = 200 nm. **(B)** TEM of dMNPs and Her-PEG-dMNPs (reduction) with scale bars = 1 µm and 200 nm. Hydrodynamic size distribution graphs of PWS-dMNP **(C)** and Her-PEG-dMNPs **(D)**. **(E)** ^1^H-NMR spectra of PWS-MNP and **(F)** PEG-dMNPs in D_2_O.

**Figure 2:**
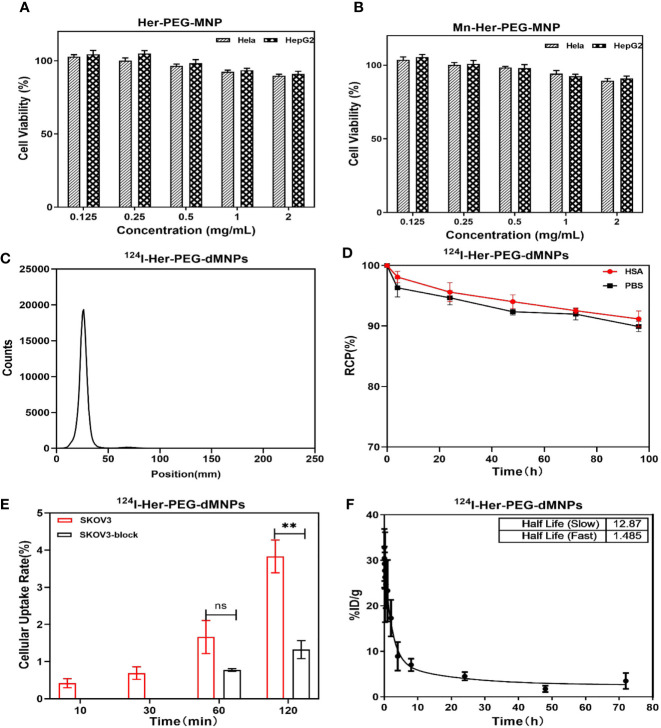
MTT assay using HeLa and HepG2 cells with **(A)** Her-PEG-dMNP and **(B)** Mn-Her-PEG-dMNP concentrations of 0.125, 0.25, 0.5, 1, and 2 mg/ml after incubation at 37°C. **(C)** Radio-TLC chromatogram of ^124^I-Her-PEG-dMNPs. **(D)** The *in-vitro* stability of ^124^I-labeled Her-PEG-dMNPs in PBS (pH = 7.4) and 5% HSA. **(E)** *In-vitro* cell uptake of ^124^I-Her-PEG-dMNPs in SKOV-3 cells. **(F)** Blood radioactivity profile of ^124^I-Her-PEG-dMNPs. Data were expressed as mean ± SEM. All the numerical data are presented as mean ± SD. *P < 0.05; **P < 0.01; ***P < 0.001 by two-way ANOVA. The symbols ** indicates statistically significant.

**Scheme 1** shows the reaction flowchart of the Her-PEG-dMNP imaging probe. Typical scanning electron microscopy (SEM) and transmission electron microscopy (TEM) imaging showed that the synthesized dMNPs and Her-PEG-DMNPs had clear structures and uniform and regular shapes and sizes (**Figures 1A, B** **)**. As shown in **Figures 1C, D** and **Figure S1**, the hydrodynamic particle size of dMNPs is about 62.25 ± 6.12 nm, and PEG-dMNP was 101.28 ± 13.63 nm, which was slightly higher than before. To construct a targeted probe for tumors with high HER2 expression, trastuzumab with an active sulfhydryl group was bound to the surface of the nanoparticles by electrophilic substitution, and the size of Her-PEG-dMNPs increased to 148.16 ± 20.04 nm. The ^1^H-NMR spectra of PEG-dMNPs in D_2_O showed an emerging peak at 3.5 ppm, which represented the –OCH_2_CH_2_O– group of the PEG molecule (**Figures 1E, F** **)**. In addition, both Her-PEG-dMNPs and Mn-Her-PEG-dMNPs showed high biocompatibility and low cytotoxicity in HepG2 and HeLa cells after being cultured for 24 h (compared with the non-toxic control). The results showed that the cell survival rate was above 95% when the concentration of both probes reached 2 mg/ml (**Figures 2A, B** **)**.

### Radiosynthesis and Quality Control of the Nanoprobe

The radiochemical yield of ^64^Cu-Her-PEG-dMNPs was 85.1%–89.6% and the specific activity was 4.77–8.25 Bq/mg, while ^124^I-Her-PEG-dMNPs had a radiochemical yield of 90%–93% and a specific activity of 7.98–13.30 MBq/mg. The final purity of both radiotracers was >98% (**Figure 2C** and **Figure S2**). The *in-vitro* stability of ^64^Cu/^124^I-Her-PEG-dMNPs in PBS and 5% HSA was evaluated by radio-TLC. The PBS (pH = 7.4) and 5% HSA had more than 89% radioactive purity at 96 h (**Figure 2D** and **Figure S2**).

### *In-Vitro* Cell Experiments

The cell uptake of ^124^I-Her-PEG-dMNPs in HER2-expressed human ovarian carcinoma SKOV-3 cell showed a higher uptake than that of ^64^Cu-Her-PEG-dMNPs in cells at 10 min, 30 min, and 1 h incubation time periods (**Figure 2E** and **Figure S2**). For SKOV-3 cells, the uptake of ^124^I-Her-PEG-dMNPs reached 0.65% ± 0.31%, 1.56% ± 0.81%, and 3.61% ± 0.74% of the collected count to the total added count (% AD/10^5^ cells) at 30, 60, and 120 min, while the uptake of ^64^Cu-Her-PEG-dMNPs was 1.55% ± 1.29%, 2.43% ± 0.51%, and 4.03% ± 0.55% AD/10^5^ cells, respectively. After blocking with the precursor, the uptake of ^124^I-Her-PEG-dMNPs at 60 and 120 min was significantly reduced (1.56 ± 0.81 *vs.* 0.72 ± 0.06 at 1 h, *P *= 0.008; 3.61 ± 0.74 *vs.* 1.24 ± 0.43 for 2 h, *P *= 0.002) (**Figure 2E**). The uptake of ^64^Cu-Her-PEG-dMNPs was significantly reduced at 1 and 2 h (2.43 ± 0.51 *vs.* 1.65 ± 0.41 for 1 h, *P *= 0.002; 4.03 ± 0.55 *vs.* 1.00 ± 0.12 for 2 h, *P *= 0.001) (**Figure S2**). These results suggest that ^124^I/^64^Cu-Her-PEG-dMNPs have specific targeting ability against HER2-expressed tumor cells.

### Pharmacokinetics of ^124^I/^64^Cu-Her-PEG-dMNPs in KM Mice

The profile of radioactivity in the blood after tail injection of ^124^I/^64^Cu-Her-PEG-dMNPs in KM healthy mice was delineated using GraphPad Prism software (**Figure 2F** and **Figure S2**). Circulating pharmacokinetics of ^124^I/^64^Cu-Her-PEG-dMNPs were evaluated using a two-compartment model. The data represent mean values, representing the percentage (ID%/g) of normal (*n* = 5) KM mice per gram of blood injection dose. The model parameters were obtained by fitting the relationship between the percentage of injected dose per gram of blood (ID%/g) and post-injection time into the following equation: Ct (%ID/g) = SpanFast * exp(*αt*) + SpanSlow * exp(*βt*), where SpanFast and SpanSlow are the *y*-intercepts of the model, and *α* and *β* are the fast and slow rate constants, expressed as the reciprocal of the time axis. In this model, the equations of ^124^I/^64^Cu-Her-PEG-dMNPs in KM mice were as follows: Ct (%ID/g) = 4.507 + 406.566 * exp(−17.81*t*) + 319.96 * exp(−0.384*t*); Ct (%ID/g) = 2.588 + 207.73 * exp(−0.466*t*) + 52.48 * exp(−0.053*t*). The half-life of ^124^I/^64^Cu-Her-PEG-dMNP distribution stage was 1.485 and 0.03892 h, respectively, while the elimination stage was 12.87 and 1.803 h, respectively.

### The Construction of the PDX Model and Immunohistochemistry Staining

All animal experiments were performed in accordance with the guidelines of the Animal Care and Use Committee of Peking University (Ethics Approval License: 2015KT08). HER2-positive patient-derived xenograft (PDX) models were built for multimodality imaging. When the tumor volume reached 0.5–1.0 cm in diameter, mice were randomly assigned. The represented immunohistochemistry results showed that the PDX tumor tissues exhibited high HER2 expression (**Figure 4C**).

### Micro-PET/CT Imaging Studies in the PDX Nude Mice

**Figure 3:**
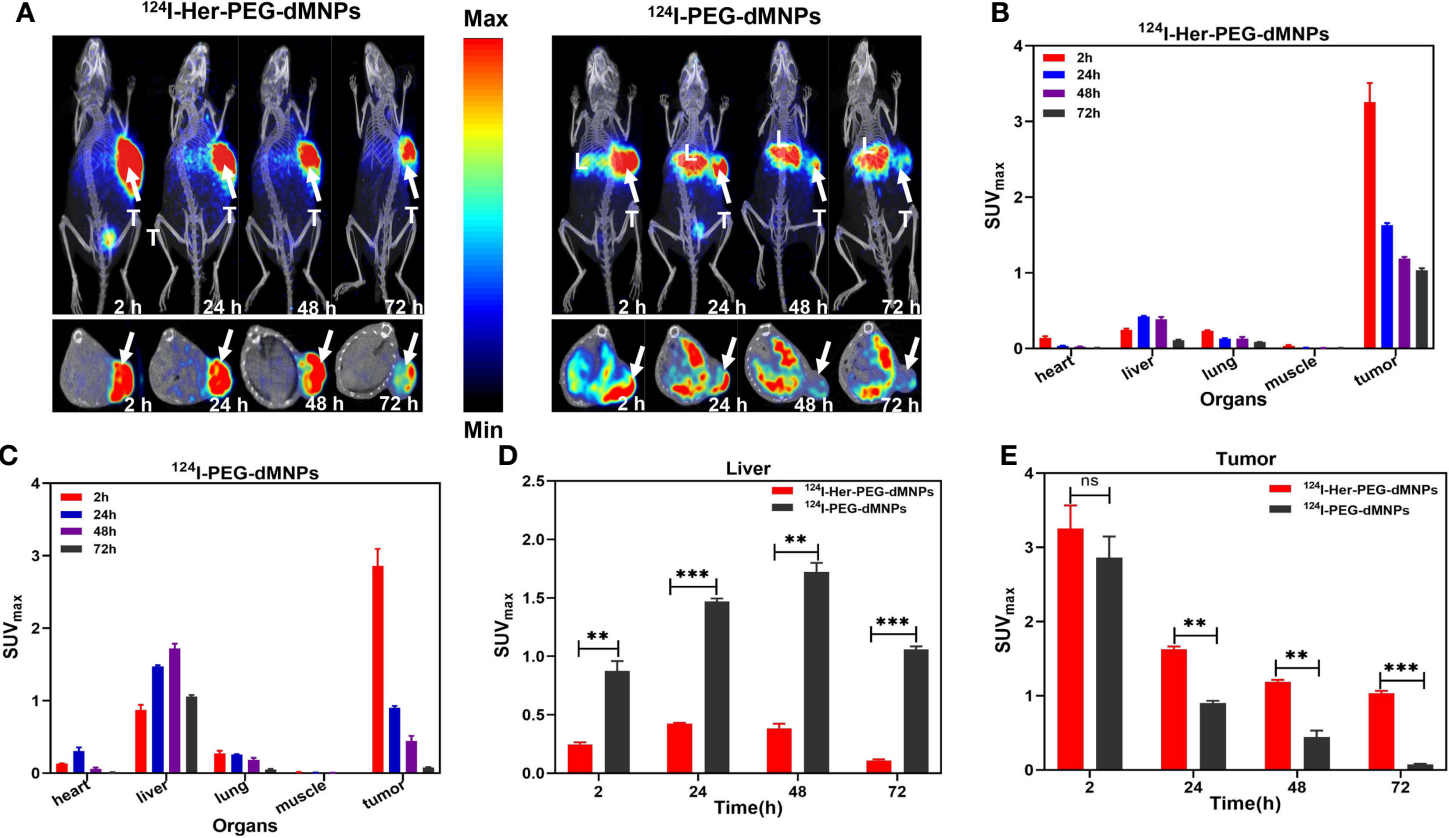
**(A)** Micro-PET/CT imaging of ^124^I-Her-PEG-dMNPs and ^124^I-PEG-dMNPs in the gastric cancer PDX model at 2, 24, 48, and 72 h post-injection (the white arrows depicted the tumors; L, liver; T, tumor). **(B, C)** The SUVmax of the organs in the PDX model based on semiquantification of ROIs. The SUVmax of the liver **(D)** and tumor **(E)** based on micro-PET/CT imaging of ROI. The data were expressed as SUVmax ± SEM. All the numerical data are presented as mean ± SD. *P < 0.05; **P < 0.01; ***P < 0.001 by two-way ANOVA. The symbols **, *** indicates statistically significant, very statistically significant.

We first used micro-PET/CT imaging to observe the accumulation of two radioactive tracers in HER2 highly expressing gastric cancer PDX model tumor site with ^124^I/^64^Cu-Her-PEG-dMNPs. In recent years, studies have shown that nanoprobes will be rapidly removed after *in-vivo* injection by human phagocytes, and their targeting function is also affected by the inevitable formation of protein crown. *In-situ* tumor injection, to some extent, can avoid such effects. The images of delayed acquisition are shown in **Figure 3A** and **Figure S3**. Both ^124^I and ^64^Cu showed excellent retention effects in the PDX model (*n* = 3) by *in-situ* injection compared with the control of the non-targeted molecular probe. ROI-based micro-PET/CT quantification analysis of those probes is shown in **Figures 3B** **, C**. The maximum standardized uptake values (SUVmaxs) of the liver were 0.24 ± 0.03, 0.42 ± 0.01, 0.38 ± 0.06, and 0.11 ± 0.01 at 2, 24, 48, and 72 h of 124I-Her-PEG-dMNPs, compared with the SUVmaxs of 0.87 ± 0.17, 1.47 ± 0.05, 1.72 ± 0.15, and 1.05 ± 0.05 at 2, 24, 48, and 72 h of ^124^I-PEGdMNPs (**Figure 3D**). The SUVmaxs of tumor sites were 3.01 ± 0.59, 1.63 ± 0.07, 1.19 ± 0.06, and 1.04 ± 0.06 of 124I-Her-PEG-dMNPs, compared with the SUVs of 2.86 ± 0.52, 0.90 ± 0.04, 0.45 ± 0.16, and 0.07 ± 0.01 of ^124^I-PEG-dMNPs (**Figure 3E**). The retention effect on the tumor was significantly different, indicating that the probe had good specificity for HER2-positive PDX tumor tissues. These results showed that the majority of ^124^I/^64^Cu-Her-PEG-dMNPs remained in the tumor at 2 h after injection, and a little part was distributed to the liver over time. The probe uptake was low in the liver and gradually decreased after 48 h, which was significantly different from the control group (^124^I-PEG-dMNPs).

### PET/MR Imaging and Analysis

**Figure 4:**
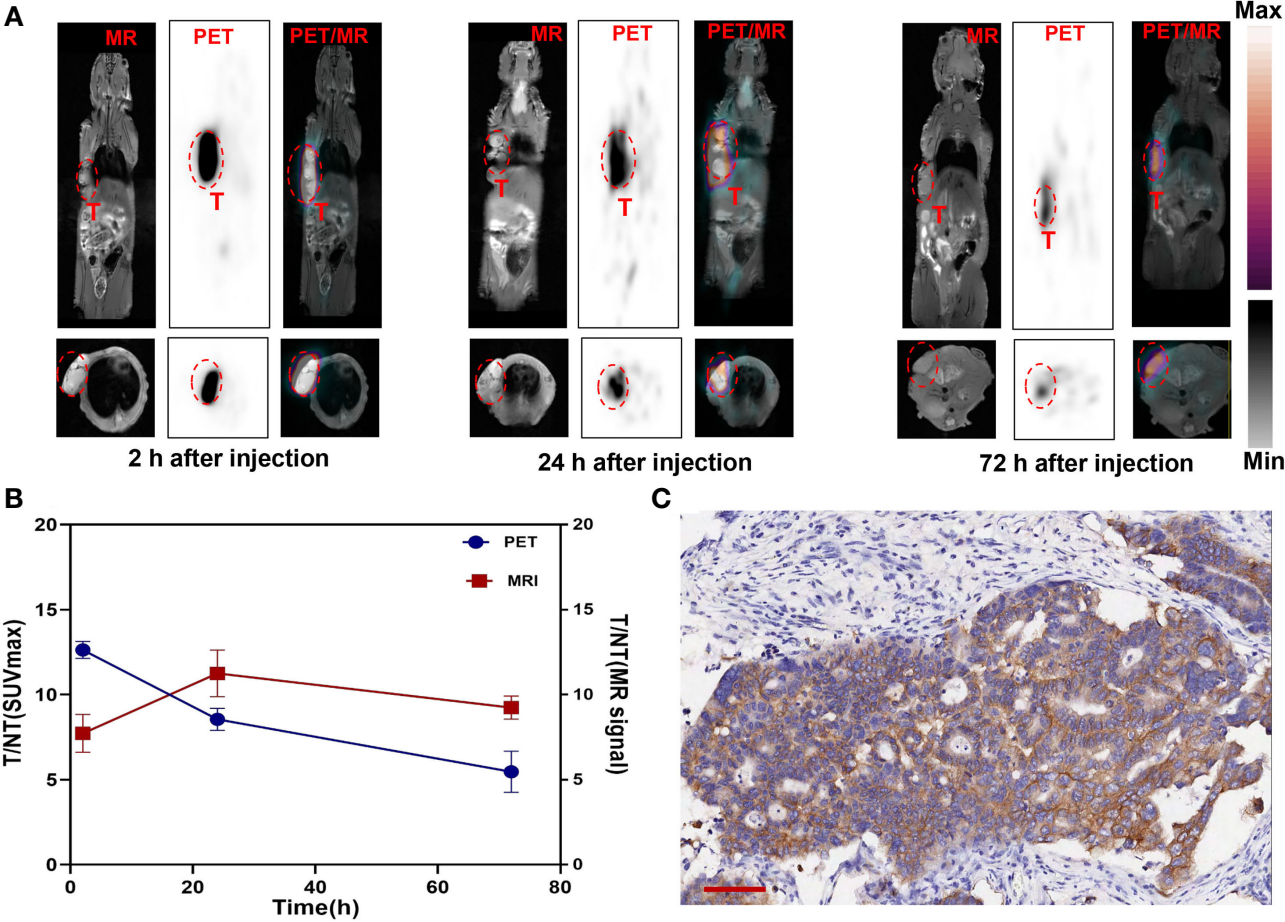
**(A)** T1-weighted MR axial images of gastric cancer PDX model at 2, 24, and 72 h post-injection Mn-Her-PEG-dMNPs (tumor site enveloped by a red dotted line). **(B)** The radioactivity distribution of PET/MRI assessed by the T/NT ratios of SUVmax and MR signal changed over time at the tumor site as determined using the ROI. **(C)** Immunohistochemical staining for gastric cancer PDX model tumor tissues. Scale bar = 100 µm.

Manganese (Mn) as a natural cell component has good biosafety and can be used in cellular and mitochondrial functions to avoid long-term side effects (33). Mn^2+^ saturation can increase the T1-weighted relaxation rate and reduce the dosage of the contrast agent (34). (^124^I, Mn)-Her-PEG-dMNPs can be used for long-term multimodal imaging monitoring over the course of treatment. Therefore, we attempted to use PET/MRI dual enhancement imaging to monitor the high tumor uptake and retention of (^124^I, Mn)-Her-PEG-dMNPs. The gastric cancer PDX tumor-bearing mice were used for imaging monitoring (*n* = 3). There were significant PET/MRI signals in the tumor site at 2 h after injection of the nanoprobe, and the signals were not concentrated in the liver or other phagocytic organs at 2, 24, and 72 h (**Figure 4A**). The T1-weighted MR signal of the tumor site slightly declined after 72 h, but the PET signal also showed strong retention at the tumor site.

Statistical analysis of the SUVmax and MR signals in the tumor site showed that the tumor/non-tumor (T/NT) values of the two signals decreased slowly (**Figure 4B**). However, the SUVmax signal and the MR signal in the liver were almost not significantly increased, which was basically consistent with the PET/MR images, indicating the strong retention effect of probes in the tumor site. This study truly realized the practical value of the theranostic functions of (^124^I, Mn)-Her-PEG-dMNPs.

## Discussion

With a half-life of 12.7 h, ^64^Cu is considered suitable for PET and tumor radionuclide therapy and can be used to radiologically label various peptides and other biologically related small molecules, monoclonal antibodies, proteins, and nanoparticles (35). With a longer half-life of 100.8 h, ^124^I is suitable for *in-vivo* monitoring of drugs with long circulation (36). ^64^Cu can label Her-PEG-dMNPs efficiently by adsorption alone. The classic iodine-labeled method with NBS was used as an oxidant to oxidize the active dihydroxyindole/indolequinone ring of Her-PEG-dMNPs for electrophilic substitution reaction labeling of ^124^I. In the field of animal models, the PDX model is a new generation of tumor model, which was established by inoculating tumor tissue of surgical resection patients into immunodeficient mice. This model retains the histological and genetic characteristics of primary tumors and maintains the heterogeneity of the tumors of patients. The pharmacodynamic results are highly correlated with clinical practice. In this study, we synthesized and characterized a novel ^124^I/^64^Cu-Mn-Her-PEG-dMNP nanoprobe and further applied it to the micro-PET/CT and PET/MR imaging in HER2 highly expressing gastric cancer PDX model.

Trastuzumab has been successfully applied in the treatment of HER2 highly expressing gastric cancer, but its potential cardiotoxicity and drug resistance limit its clinical use (10). We have tentatively designed a novel nanoprobe to be coupled with trastuzumab to solve these problems. This nanoprobe fused active targeting and passive targeting, which can greatly improve the targeting efficiency and retention time in the tumor site and reduce the biological half-life of monoclonal antibodies. In addition, the melanin nanoparticles used in this study have photothermal treatment functions, and they can also label therapeutic nuclides to construct integrated theranostics probes to reduce drug resistance during long-term treatment.

The dMNPs were synthesized by the oxidation and self-polymerization of dopamine in a simple method. We selected radionuclide ^124^I and ^64^Cu to construct a micro-PET/CT imaging nanoprobe for matching the pharmacokinetic distribution of Her-PEG-dMNPs *in vivo*. Melanin has a strong chelating property for a variety of metal ions, including Cu^2+^, Fe^3+^, and Mn^2+^. The melanin nanoparticles can perform metal adsorption and nuclide labeling without the introduction of chelating groups, which greatly simplifies the preparation process and reduces the possible probe heterogeneity. Mn^2+^ is a trace element in the human body and can be fully metabolized in living organisms (33). In contrast, although Gd^3+^ has a higher T1-weighted MR relaxation rate than Mn^2+^, its potential brain toxicity remains an important issue (37).

In this study, the labeling and purification process of ^124^I-Her-PEG-dMNPs took only 5 min with 98% radiochemical yield, suggesting a more convenient labeling synthesis with higher yield. However, the labeling and purification process of ^64^Cu-Her-PEG-dMNPs took 1 h, and the average labeling rate was lower than ^124^I-Her-PEG-dMNPs. After 96 h of incubation, the average radiochemical yield reached 89% of ^124^I-Her-PEG-dMNPs and ^64^Cu-Her-PEG-dMNPs, which proved that both radioactive probes have good stability *in vitro*. In the cell uptake experiment, the uptake of ^124^I/^64^Cu-Her-PEG-dMNPs in SKOV-3 cells, which have high HER2 expression, could be blocked by excessive Her-PEG-dMNPs. While both probes showed preliminary specificity for HER2 in SKOV-3 cells, ^124^I-Her-PEG-dMNPs showed superior contrast due to better matching of full-length antibody pharmacokinetics, which has better application prospects. The micro-PET/CT and PET/MRI of different nanoprobes indicate that the ^124^I-Her-PEG-dMNP probe has high specificity for the PDX model and also conforms to the EPR effect of nanoparticles (38). In MRI imaging, the magnetic resonance signal of the tumor site was significantly enhanced with the passage of time, which proved that Mn-Her-PEG-dMNPs had a good contrast effect. At present, the clinical application of MRI-targeted contrast agents have been rarely reported, which is related to the poor sensitivity of traditional contrast agents, and only excessive contrast agent injection can obtain the enhancement effect of image visibility. The new multimodal imaging probe constructed in this study was based on the high drug loading rate of nanoparticles, which significantly improved the load of the MRI contrast agent, and was expected to provide guidance for further development of magnetic resonance-targeted contrast agents.

## Conclusions

We developed novel nanoprobes targeting a HER2 expression gastric cancer PDX model with micro-PET/CT and PET/MRI imaging to improve the retention time of monoclonal antibodies in tumor sites, reduce cardiac toxicity, and monitor the therapeutic effect in real time through multimodal imaging. Then, the safety of the nanoprobes was verified by cytotoxicity characterization, and its specificity and metabolism were evaluated by *in-vitro* cell uptake and pharmacokinetics. These results support the possibility of using the nanoprobe for the diagnosis of theranostics in gastric cancer.

## Data Availability Statement

The raw data supporting the conclusions of this article will be made available by the authors, without undue reservation.

## Ethics Statement

The animal study was reviewed and approved by Peking University Cancer Hospital Animal Care and Use Committee.

## Author Contributions

ZY and HZ designed the study. XY, XG, and FW analyze data. LW and LX completed the experiment and wrote the paper together. All authors discuss the results and critique of the manuscript. All authors contributed to the article and approved the submitted version.

## Funding

This work was supported, in part, by the National Natural Science Foundation of China (Nos.81960538, 81671733 and 81871386), the Science and Technology Foundation of Guizhou (No. [2019] 1021 and ZK[2021]471), Beijing Municipal Administration of Hospitals-Yangfan Project (ZYLX201816), Beijing Millions of Talent Projects A level funding (2019A38), Science Foundation of Peking University Cancer Hospital (2021-17), and open project funded by Key laboratory of Carcinogenesis and Translational Research) Ministry of Education/Beijing (2019 Open Project- 06).

## Conflict of Interest

The authors declare that the research was conducted in the absence of any commercial or financial relationships that could be construed as a potential conflict of interest.

## Publisher’s Note

All claims expressed in this article are solely those of the authors and do not necessarily represent those of their affiliated organizations, or those of the publisher, the editors and the reviewers. Any product that may be evaluated in this article, or claim that may be made by its manufacturer, is not guaranteed or endorsed by the publisher.
