# Supplementary material for: Multimodal Imaging Technology Effectively Monitors HER2 Expression in Tumors Using Trastuzumab-Coupled Organic Nanoparticles in Patient-Derived Xenograft Mice Models
Source: Front Oncol. 2021 Nov 17;11:778728. doi: 10.3389/fonc.2021.778728 (PMC8637767; doi:10.3389/fonc.2021.778728)
Supplement: Supplementary file 1 [file DataSheet_1.docx]

**Suppoting information**

**Multimodal imaging technology effectively monitors HER2 expression in tumors using** **trastuzumab-coupled organic nanoparticles in patient-derived xenografts mice models**

Li Wen^#1,2^, Lei Xia^# 2^, Xiaoyi Guo^2^, Hai-Feng Huang^3^, Feng Wang^2^, Xian-teng Yang*^1,3^, Zhi Yang*^1,2^, Hua Zhu*^1, 2^

**
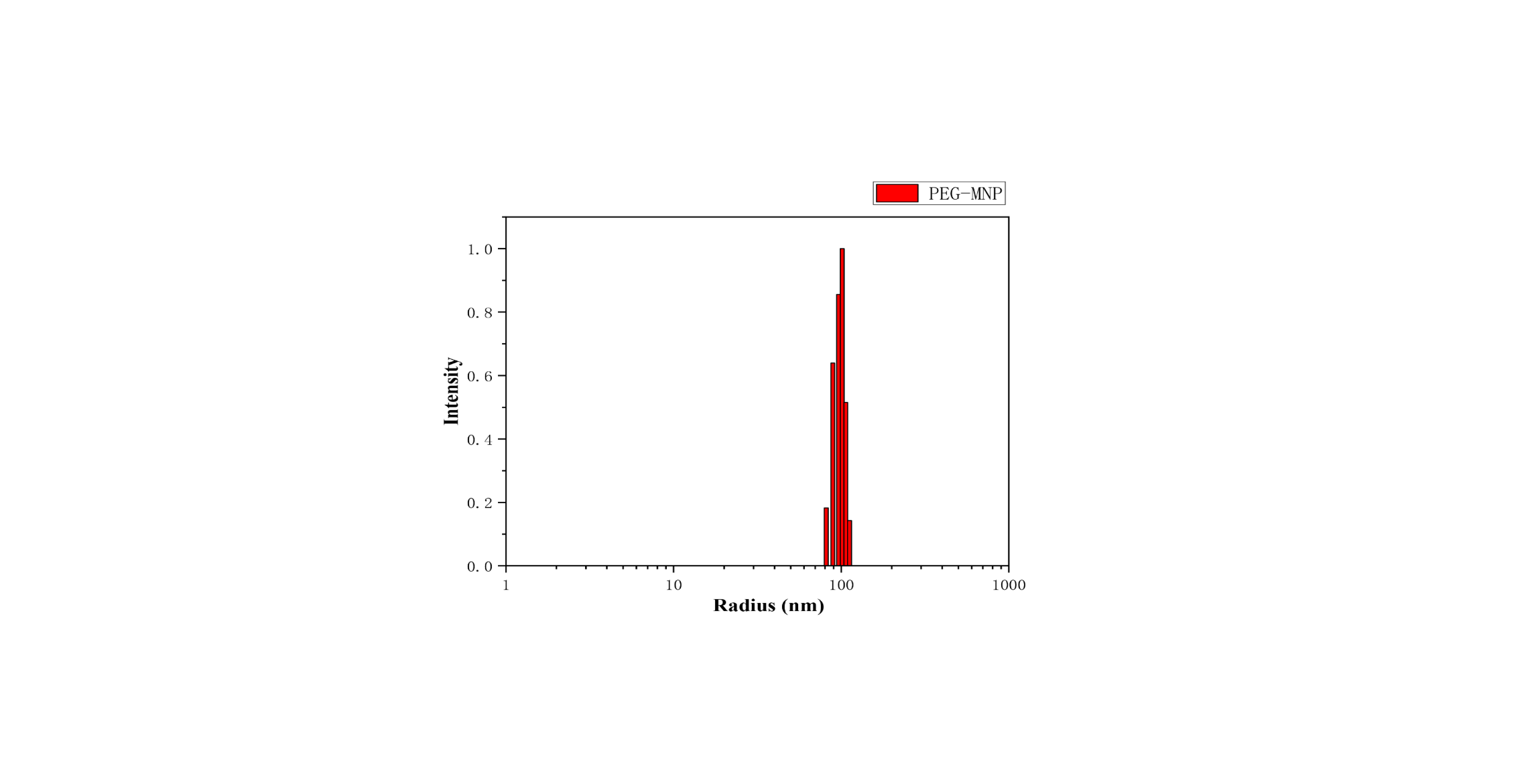
**

**Figure 1 │** Hydrodynamic size distribution graphs of PEG-dMNPs.


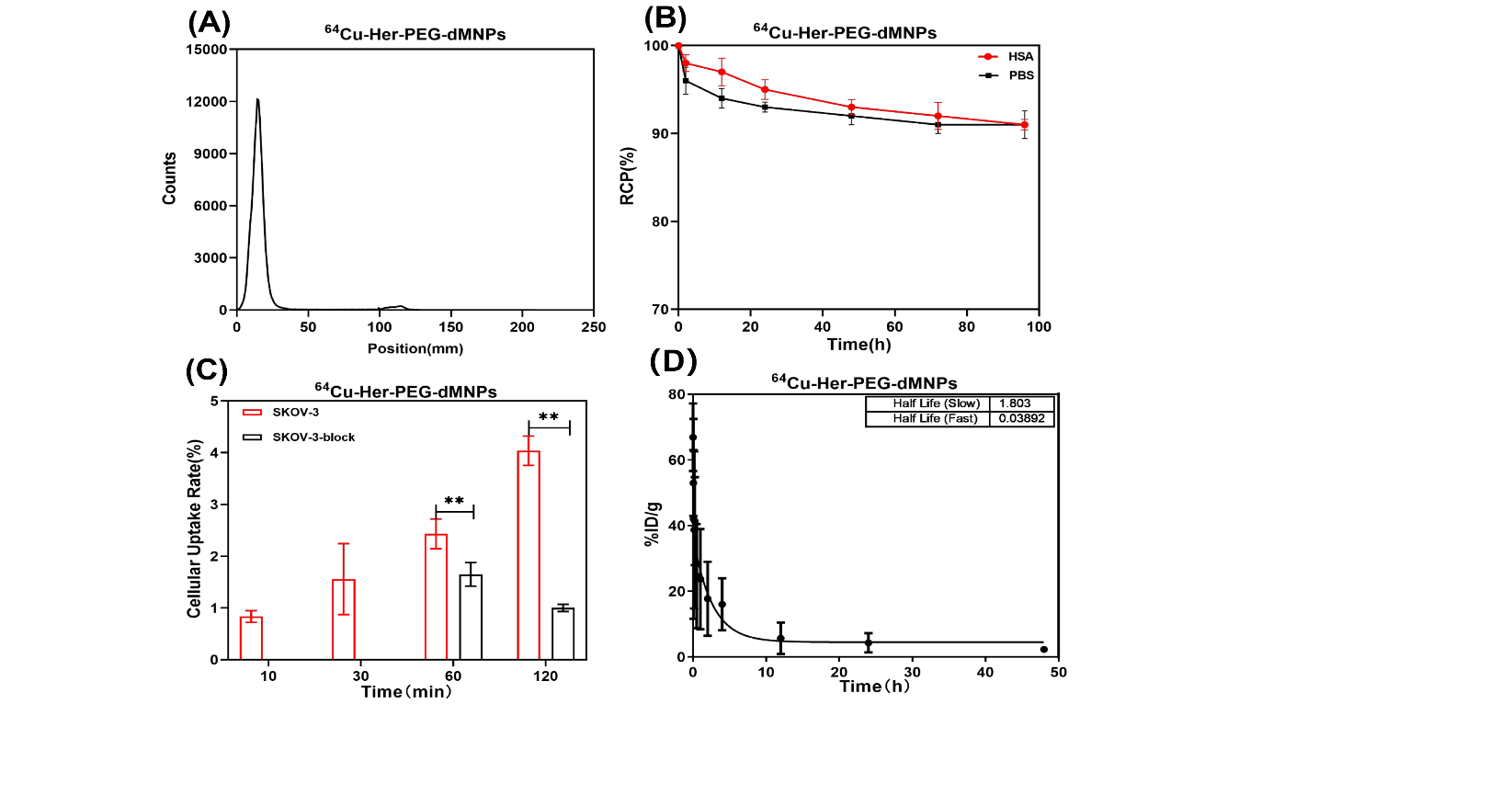


**Figure 2 │(A)** Radio-TLC chromatogram of ^64^Cu-Her-PEG-dMNPs; **(B)** The in vitro stability of ^64^Cu labeled Her-PEG-dMNPs in PBS (pH = 7.4) and 5% HSA; **(C)** In vitro cell uptake of ^64^Cu-Her-PEG-dMNPs and ^124^I-Her-PEG-dMNPs in SKOV-3 cells. **(D)** Blood radioactivity profile of ^64^Cu-Her-PEG-dMNPs. All the numerical data are presented as mean ± SD. *P < 0.05; **P < 0.01; ***P < 0.001 by two-way ANOVA.


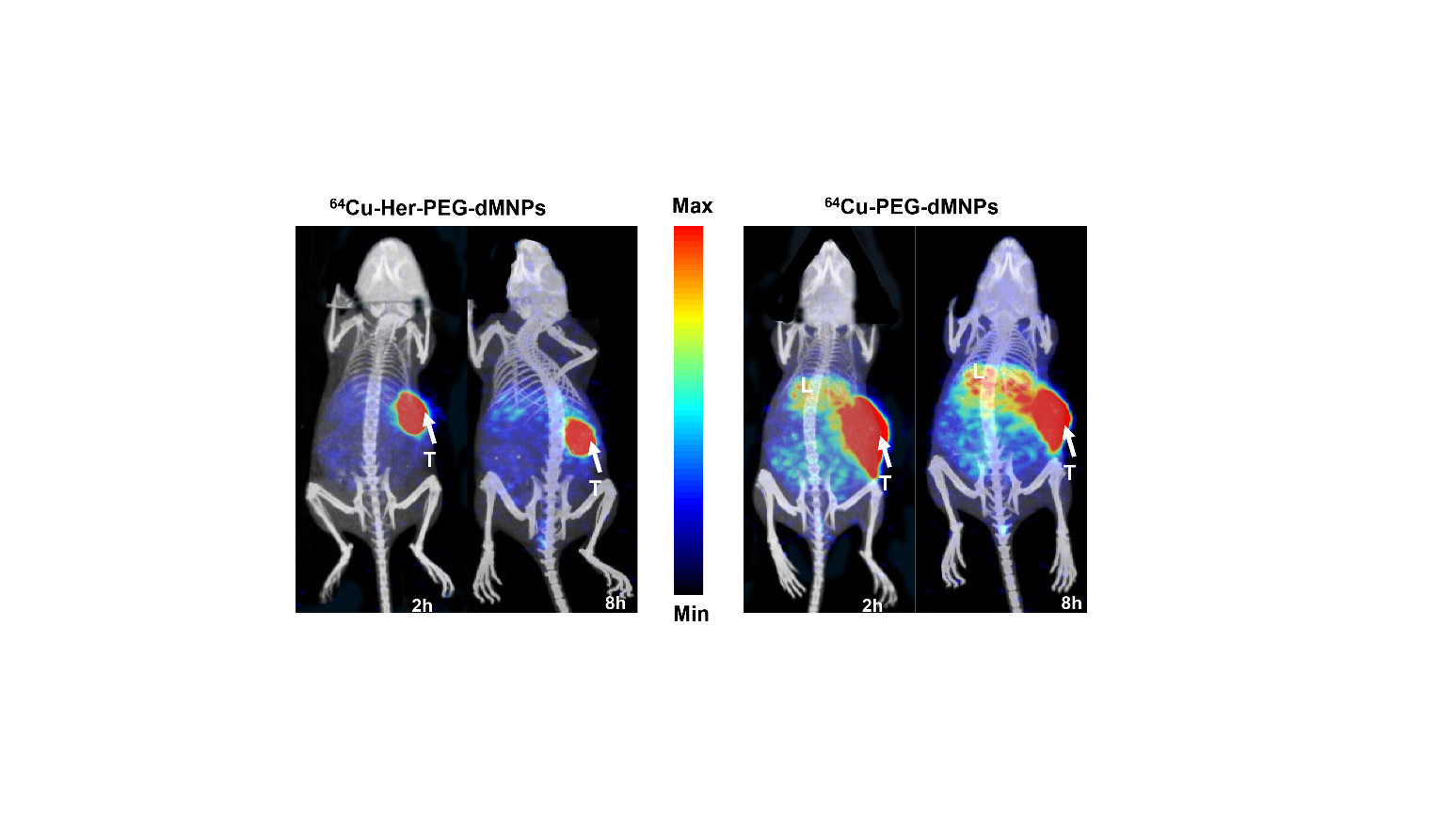


**Figure 3│**Micro-PET/CT imaging of ^64^Cu-Her-PEG-dMNPs and ^64^Cu-PEG-dMNPs in BALB/c nude mice bearing gastric cancer PDX model at 2 h and 8 h post-injection (The white arrows depicted the tumors, L-Liver, T-tumor).
